# A Case Report of Acute Prostatitis Secondary to Use of P-valve Condom Catheter During Cave Diving

**DOI:** 10.5811/cpcem.2021.7.52639

**Published:** 2021-09-09

**Authors:** Ashley Barash, Evan Stern, Robyn Hoelle

**Affiliations:** * North Florida Regional Medical Center, Department of Graduate Medical Education, Gainesville, Florida; † UCF/HCA Healthcare GME Consortium of North Florida, Department of Emergency Medicine, Gainesville, Florida

**Keywords:** case report, acute bacterial prostatitis, cave diving, Aeromonas

## Abstract

**Introduction:**

Acute bacterial prostatitis is characterized by acute inflammation of the prostate gland accompanied by the presence of pain and other urinary tract or systemic symptoms. Prostatitis is a relatively common disease of the urinary tract in men, However, this case reports a man diagnosed with acute bacterial prostatitis with an unusual presentation, as well as an unusual pathogen and a unique mechanism of colonization.

**Case Report:**

A 52-year-old male with no past medical history presented to our facility for right-sided buttock pain associated with dysuria, diarrhea, and perianal burning. The patient was diagnosed with sepsis secondary to acute bacterial prostatitis, and the pathogen identified in his urine was *Aeromonas hydrophila/A. caviae*. His disease process was later recognized as a complication of the use of a P-valve condom catheter while freshwater cave diving.

**Conclusion:**

This is the first documented case of prostatitis as a result of the use of a P-valve condom catheter while diving. Furthermore, the pathogen identified is of particular interest as there are very few documented cases of urosepsis secondary to *Aeromonas hydrophila* or *A. caviae*.

## INTRODUCTION

Prostatitis is the third most common urinary tract disease in men. Acute bacterial prostatitis is characterized by acute inflammation of the prostate gland accompanied by the presence of pain and urinary tract symptoms. It most commonly occurs in men in two predominant groups: those 20–40 years old and those >70. The most common pathogen identified in acute bacterial prostatitis is *Escherichia Coli*; however, *Neisseria gonorrhea* and *Chlamydia trachomatis* need to be considered in those who are sexually active.^1^ While prostatitis is a relatively common disease of the urinary tract, this case report of a man with acute bacterial prostatitis is anything but ordinary. We describe a 52-year-old male with an unusual presentation diagnosed with acute bacterial prostatitis colonized from a condom catheter that he used while cave diving in freshwater.

## CASE REPORT

A 52-year-old male with no past medical history presented to our emergency department (ED) for right-sided buttock pain that started in the middle of the night. He also described dysuria, which progressed to perianal burning associated with an episode of diarrhea and right-sided buttock pain. His pain was severe in nature, waxing and waning and non-radiating. The patient reported freshwater cave diving in north-central Florida 15 hours prior to onset of symptoms. He reported diving to a depth of 100 feet with a slow assent. He experienced no adverse symptoms and reported feeling normal prior to going to bed the night before.

On initial examination, the patient was tachycardic with a heart rate of 109 beats per minute. Remaining vital signs were within normal limits, including a temperature of 37.1° Celsius. He appeared in distress secondary to pain. His skin was pale and diaphoretic. His abdominal exam was benign without tenderness to palpation, rebound or guarding. There was tenderness to palpation over the right buttock approximately halfway between the posterior superior iliac spine and ischial tuberosity. No obvious wounds or traumatic injuries were present, and there was no erythema, warmth or swelling to indicate cellulitis or abscess. Repeat vital signs approximately 3.5 hours after initial evaluation remained unchanged with the exception of a rising temperature to 37.9°C.

Labs in the ED were significant for white blood cell count (WBC) of 22.7 thousand per millimeter^3^ (K/mm^3^) (reference 4.5–11 K/mm^3^) and lactic acid of 4.8 millimoles per liter (mmol/L) (0.4–2.0 mmol/L). Blood urea nitrogen (BUN) was 16 milligrams per deciliter (mg/dL) (7–18 mg/dL), and creatinine (Cr) was 1.32 mg/dL (0.60–1.30 mg/dL). Urinalysis was significant for small protein mg/dL (reference: negative); small ketones mg/dL (reference: negative); large leukocyte esterase (reference: negative); too numerous to count per high power field (HPF) WBCs (reference: 0–5 per HPF); 30–40 per HPF red blood cells; and moderate hemoglobin (reference: negative).

Computed tomography of the abdomen and pelvis was obtained given concern for abdominal, pelvic or lumbar etiology of the pain and demonstrated “hazy inflammation involving the prostate, rectosigmoid colon and seminal vesicles, which likely represents a prostatitis with possibly associated proctitis. The seminal vesicles also appear inflamed.” The patient received a 30 milliliter per kilogram (mL/kg) normal saline fluid bolus and intravenous cefepime. He was admitted to the inpatient service for continued treatment of sepsis likely secondary to prostatitis/colitis. Urology was consulted as an inpatient.

On day one of hospitalization, he developed acute urinary retention requiring Foley catheter placement. Prostate-specific antigen returned elevated at 8.36 nanograms per milliliter (ng/mL) (reference 0.0–4.4 ng/mL) and urine cultures grew *Aeromonas hydrophila/A caviae*. Urine culture was unable to provide further speciation distinguish between *Aeromonas hydrophila* and *A caviae*. Cefepime was continued for four days based on antibiotic sensitivities with significant clinical improvement. The Foley catheter was removed on day three and the patient was able to void successfully. His WBC count improved to 5.9 K/mm^3^, BUN was 7 mg/dL, and Cr was 0.92 mg/dL. He was discharged on day four with ciprofloxacin 500 milligrams (mg) twice a day for two weeks.

## DISCUSSION

This is a case where a patient presented with a chief complaint of “right buttock pain.” Acute bacterial prostatitis can present with a variety of clinical symptoms to include urinary symptoms, suprapubic, rectal or perianal pain, painful ejaculation, hematospermia, and painful defecation. It often also presents with systemic symptoms such as fever, chills, nausea, emesis, and malaise. While this patient did mention an episode of dysuria associated with perianal burning, his primary complaint was pain located in the right buttock. The physical examination findings for prostatitis can vary but often include an enlarged, tender, or boggy prostate. There may also be abdominal distention indicative of a distended bladder secondary to obstructive pathology.^2^

**CPC-EM Capsule**

What do we already know about this clinical entity?

*Acute bacterial prostatitis is characterized by acute inflammation of the prostate gland accompanied by localized pain and urinary tract symptoms.*

What makes this presentation of disease reportable?

*We describe a patient diagnosed with acute bacterial prostatitis after use of a condom catheter during underwater cave diving, with an aquatic pathogenic bacteria,* Aeromonas Hydrophila.

What is the major learning point?

*When external water flows in a retrograde fashion into the tubing of a P-valve condom-catheter, divers are at risk of urinary tract infection.*

How might this improve emergency medicine practice?

*Understanding the mechanism of anerobic bacteria colonization and specific pathogens for which cave divers are at risk will help guide management in the emergency department.*

This patient’s physical exam was remarkable primarily for tenderness to palpation in the right gluteal region. There are several possibilities as to why the pain localized to this region. One theory is based on information regarding pain associated with chronic nonbacterial prostatitis/chronic pelvic pain syndrome (or Type III prostatitis). Chronic pelvic pain syndrome (CPPS) is described as a pain, pressure or discomfort in the pelvic region, perineum or genitalia that occurs in the absence of bacteria. The pain associated with CPPS is often multifactorial and involves more than one body system. There is evidence of a mechanical relationship between various structures within these anatomical regions; for example, thoracolumbar dysfunction is known to have referred pain into the testicular region. While CPPS occurs in the absence of uropathogenic bacteria, it is possible that acute bacterial prostatitis has similar physical manifestations resulting in a referred pain mechanism to the gluteal region.^3^

While there are several documented cases of urinary tract infections following scuba diving, most documented cases identified the common pathogen as *Pseudomonas aeroguinosa,* most likely because the *Pseudomonas* species thrives in both freshwater and saltwater environments.^4^ Our patient, however, presented with urosepsis secondary to *Aeromonas*, a Gram-negative bacteria that primarily thrives in aquatic environments. There are 36 species within the genus with 19 identified pathogens in humans. Ninety-five percent of the pathogens identified in humans were identified as only four species: *Aeromonas caviae* (37.26%), *Aeromonas dhakensis* (23.49%), *Aeromonas veronii* (21.54%), and *Aeromonas hydrophila* (13.07%). In humans, *Aeromonas* has primarily been identified in cases of gastroenteritis, bacteremia and wound infections, and *A hydrophila,* specifically*,* which was identified as the source of infection in this case, has been identified more in extra-intestinal infections.^5^,^6^

Examples of urosepsis secondary to *Aeromonas* are limited and were in association with patients who had undergone a recent prostatic biopsy. In a study that assessed complications of transrectal prostate biopsy, urosepsis occurred in 10 patients of whom only one had positive blood culture for *Aeromonas*.^7^ Importantly, *Aeromonas* present in extraintestinal samples had high rates of drug resistance according to one study, including those antibiotics commonly used to treat urinary tract infections.^6^ Aside from cases associated with prostate manipulation such as transrectal prostate biopsy, we could identify no cases that report colonization secondary to scuba diving. This case is unique both in terms of the uncommon pathogen and the mechanism of colonization. As mentioned, this patient presented 15 hours after freshwater cave diving. He later revealed that during long cave dives, he uses a P-valve condom catheter to urinate. We suspect that this P-valve condom catheter contributed to his mechanism of colonization.

P-valve condom catheters allow urination outside a dry suit during prolonged dives. There are two types of P-valve systems: balanced and unbalanced. Balanced P-valves have a one-way valve and a balancing chamber that allows air to enter catheter tubing, equalizing the pressure within the tubing equal to the pressure at any given depth. Unbalanced tubes have a manual valve that must be opened to release urine out of the catheter and should be primed prior to descent to prevent squeeze during descent. The unbalanced P-valve system can allow external water to flow in a retrograde fashion into the catheter tubing if the manual valve is left open during the dive or when it is opened to urinate.

Our patient used an unbalanced P-valve system and stated that the valve was open throughout the dive. This in turn allowed a constant retrograde flow of water up through the tubing and into the condom catheter. It is therefore likely that bacterial colonization occurred via this mechanism. Our literature search failed to demonstrate any other similar cases of prostatitis secondary to use of a P-valve condom catheter. One case report identified a 39-year-old professional diver who suffered recurrent cystitis and nephrolithiasis secondary to a P-valve condom catheter. The report, which identified as a case of cystitis and nephrolithiasis due to *Pseudomonas aeruginosa*, demonstrated a failure in the P-valve allowing constant retrograde flow into the valve.^4^

## CONCLUSION

While acute bacterial prostatitis is a common disease with defined common pathogens, this is the first documented case of prostatitis associated with the use of a P-valve condom catheter while diving. Furthermore, the pathogen identified is of interest as there are very few documented cases of urosepsis secondary to *Aeromonas hydrophila/A caviae*. We describe a unique adverse event associated with cave diving that emergency physicians should be prepared to evaluate, diagnose, and treat.
